# Experimental Investigation of Surface Roughness and Material Removal Rate in Wire EDM of Stainless Steel 304

**DOI:** 10.3390/ma16031022

**Published:** 2023-01-23

**Authors:** Noha Naeim, Mona A. AbouEleaz, Ahmed Elkaseer

**Affiliations:** 1 Department of Production Engineering and Mechanical Design, Faculty of Engineering, Port Said University, Port Fuad 42526, Egypt; 2 Production Engineering and Mechanical Design Department, Faulty of Engineering, Mansoura University, Mansoura 35516, Egypt; 3 Institute for Automation and Applied Informatics, Karlsruhe Institute of Technology, 76344 Eggenstein-Leopoldshafen, Germany; 4 Department of Mechanical Engineering, Faculty of Engineering, The British University in Egypt (BUE), El-Sherouk City 11837, Egypt

**Keywords:** WEDM, stainless steel 304, MRR, surface roughness, statistical analysis

## Abstract

Its unexcelled mechanical and physical properties, in addition to its biocompatibility, have made stainless steel 304 a prime candidate for a wide range of applications. Among different manufacturing techniques, electrical discharge machining (EDM) has shown high potential in processing stainless steel 304 in a controllable manner. This paper reports the results of an experimental investigation into the effect of the process parameters on the obtainable surface roughness and material removal rate of stainless steel 304, when slotted using wire EDM. A full factorial design of the experiment was followed when conducting experimental trials in which the effects of the different levels of the five process parameters; applied voltage, traverse feed, pulse-on time, pulse-off time, and current intensity were investigated. The geometry of the cut slots was characterized using the MATLAB image processing toolbox to detect the edge and precise width of the cut slot along its entire length to determine the material removal rate. In addition, the surface roughness of the side walls of the slots were characterized, and the roughness average was evaluated for the range of the process parameters being examined. The effect of the five process parameters on both responses were studied, and the results revealed that the material removal rate is significantly influenced by feed (*p*-value = 9.72 × 10^−29^), followed by current tension (*p*-value = 6.02 × 10^−7^), and voltage (*p*-value = 3.77 × 10^−5^), while the most significant parameters affecting the surface roughness are current tension (*p*-value = 1.89 × 10^−7^), followed by pulse-on time (1.602 × 10^−5^), and pulse-off time (0.0204). The developed regression models and associated prediction plots offer a reliable tool to predict the effect of the process parameters, and thus enable the optimizing of their effects on both responses; surface roughness and material removal rate. The results also reveal the trade-off between the effect of significant process parameters on the material removal rate and surface roughness. This points out the need for a robust multi-objective optimization technique to identify the process window for obtaining high quality surfaces while keeping the material removal rate as high as possible.

## 1. Introduction

Materials processing technologies play an important role in producing varieties of products for a wide range of engineering applications [1], especially, recent engineering applications necessitate the development of functional parts composed of advanced materials with superior mechanical and thermal properties, so-called superalloys [2]. However, processing such superalloys to produce high precision features with a tight tolerance and high accuracy is not straightforward, and in most cases, robust manufacturing techniques, more likely non-conventional ones, have to be utilized [3].

In mechanical-based chip removal processes, a large amount of energy is exerted to remove undesirable chips which must be discarded [4]. Nevertheless, the large body of machining energy ends in unwanted heat, which could cause problems with surface integrity, surface cracking, and distortion [5]. In addition, residual stresses and burrs could also be generated during the machining process and predominantly require further post-processing steps [6]. In general, it is worth stating that superalloys with high strength and wear resistance are difficult to be machined using traditional machining processes, such as turning, drilling, shaping, and milling [7]. This limitation has led to the development of non-traditional machining processes with high potential competence to fabricate components with complex features in superalloy materials besides their superior mechanical and thermal properties [8].

Non-traditional manufacturing techniques can be defined as a cluster of processes that are used to remove surplus material by several techniques based on mechanical, thermal, electrical, or chemical interaction, or even blends of these energies without using sharp cutting tools to remove chips, similar to those used in traditional mechanical manufacturing processes [9]. A number of non-traditional machining processes have been developed to meet the requirements of the final parts to be produced [9]. Realistically, compared to conventional machining techniques, non-traditional machining processes are usually associated with low productivity and high specific energy consumption compared with traditional machining techniques [10,11]. Nevertheless, when the appropriate processing parameters are rigorously applied, non-traditional machining can lead to the generation of precise features with a high accuracy and surface quality [12]. Moreover, some processes, such as electrical discharge machining (EDM), have been proven to produce complex features with a tight tolerance, high accuracy, and, to some extent, an acceptable throughput [13].

**Figure 1:**
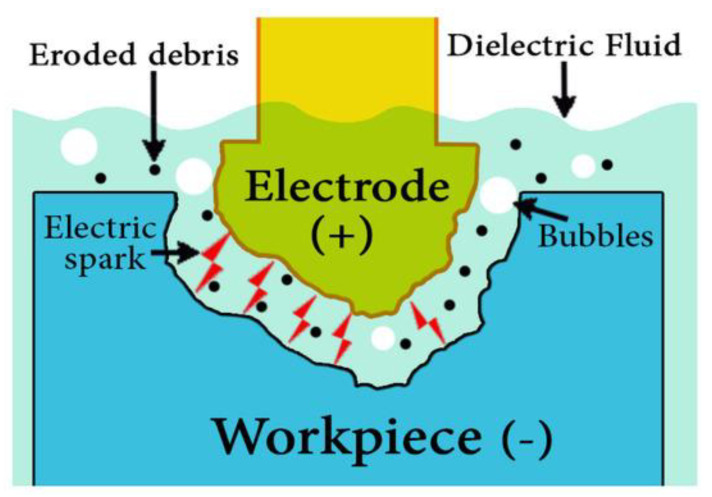
Working principles of the EDM process [16].

EDM is one of the earliest and most widely used non-traditional machining processes in the production industries because of its ability to machine hard materials [14], complex shapes [15], precise and irregular shapes for forging [15], press tools [15], extrusion dies [15], and complex internal shapes in aerospace and medical applications that need high surface finishes and dimensional accuracy [15]. The main different attribute of EDM when compared to traditional machining processes is that the EDM technique uses a thermoelectric phenomenon to corrode undesired materials from the work piece using a sequence of discrete electrical sparks between the work piece and the electrode tool [14], see Figure 1.

**Figure 2:**
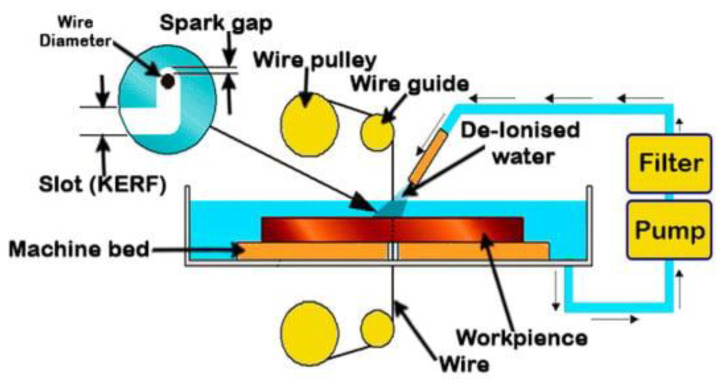
Working principles of the wire EDM process [19].

As an improved modification of general EDM, in wire EDM (see Figure 2), the material removal process does not include contact between the tool, a stretched wire in this case, and the work material. However, similar to the generic EDM techniques, the erosion is conducted by a series of sparks between both electrodes [17]. The electrical discharge occurs between the two electrodes in the presence of dielectric fluid over a small distance called the “gap.” The workpiece is called a cathode, while the wire electrode is termed an anode. The governing factors are the intensity of the spark, the electric pulse parameters, the material combination of the workpiece and wire, and the dielectric fluid [18].

A number of related works have been reported in the literature. Raju et al. [20] studied the effects of pulse-on time (T_ON_), peak current (IP), servo voltage (SV), and wire tension (WT) on the wear behavior of 316 L stainless steel using a wire electric discharge machine (WEDM). Sliding wear tests are carried out using a pin-on-disc device. The microstructure of the specimens was evaluated using SEM and XRD. The results showed that the most influential parameters affecting wear resistance were determined to be T_ON_ and IP. The wear resistance stayed constant for the comparable values of the products of T_ON_ and IP, and as the products of T_ON_ and IP decreased, the wear resistance increased. The microhardness and wear resistance are found to be high when compared to other machining conditions and as received in 316L SS material at lower levels of pulse-on-time and IP. Moreover, when the amount of discharge energy increases, a high rate of material removal is achieved.

Gowthaman et al. [21] performed an experimental study of the effect of WEDM parameters, such as pulse-on-time, pulse-off-time, and servo voltage, on the material removal rate and surface roughness of AISI 4340 alloy steel using an “Electronica Spring Cut-WEDM Machine”, in which zinc-coated brass wire with a diameter of 0.25 mm was used as an electrode. Experimental results showed that increasing the pulse duration, wire speed, and open circuit voltage increased the metal removal rate. In addition, the surface roughness increases with an increase in pulse-on-time and discharge energy. Increasing the pulse-off time led to increased surface roughness. Moreover, increasing the pulse-off time and servo voltage decreased the material removal rate significantly.

Chaudhary et al. [22] examined the effect of a wide range of wire tension on surface roughness, kerf width, material removal rate, recast layer hardness, and surface microhardness for AISI 304. Pulse-on-time, pulse-off-time, current, and dielectric fluid are taken as fixed parameters. The experimental work was performed on Steer Corporation’s DK7712 CNC-WEDM machine. De-mineralized (DM) water was used as a dielectric fluid. The wire tension was varied through a precision gauge mounted on a specially devised fixture. Molybdenum wire with a 0.18 mm diameter was used as an electrode. Results showed that SR decreased with increasing wire tension because wire vibrations decreased. MRR improved slightly by increasing wire tension due to wire tightness and straightness. Wire tension did not have a significant effect on kerf width. However, the kerf width has not changed with changes in wire tension. Furthermore, as the wire tension increased, the recast layer and surface microhardness decreased [23].

Sen et al. [24] conducted an experimental investigation of the Wire EDM performance of AISI 304 stainless steel with the aid of the Taguchi L16 orthogonal array. The experimental work used a brass wire with a diameter of 0.25 mm and deionized water as a dielectric fluid. The experimental study included five process parameters: pulse-on time, pulse-on time, peak current, wire feed, and wire tension. The authors found that the most crucial factor influencing machining characteristics is pulse rate. Moreover, the peak current and pulse-off time strongly affected the surface roughness. 

Van Sy [25] used multi-response optimization to assess the performance of machining parameters in the WEDM process on both surface roughness and geometric accuracy. The study examined the WEDM parameters, such as wire tension, wire speed, pulse-on/pulse-off time, and peak current, to demonstrate their effect on both dimensional accuracy and surface quality in machining a die-angular model and the multi-objective optimization technique to realize optimal processing parameters for machining stainless steel (grade 304) products. The study proved that the discharge current and pulse frequency significantly affected the geometric accuracy of the acute corner and decreased significantly when machining the larger corners or arcs. Moreover, the wire speed and wire tension also affect the geometric accuracy of the larger corners. 

Kumar et al. [26] investigated the WEDM of steel 304 using a RSM design called a face-centered design. The study included distinct WEDM parameters such as release flow, wire speed, wire pressure, dielectric stream rate, beat on time (T_ON_), and beat off time (T_OFF_) and addressed their effect on the yield parameters (material removal rate (MRR) and surface roughness). The optimal set of process parameters has also been predicted to maximize the MRR and surface finish.

Mathew et al. [27] analyzed the experimental work of WEDM on AISI-304 using Taguchi grey relational analysis, considering distinguished machining parameters, such as pulse-on/pulse-off time, servo voltage, wire tension, wire feed, and dielectric pressure. Moreover, they addressed the optimum combination of the WEDM parameters as the pulse-on time level 1, pulse-off time level 2, servo voltage level 1, wire tension level 3, wire feed level 3, and water pressure level 3. 

Lingadurai et al. [28] performed experimental work to investigate the effects of machining parameters on WEDM for AISI 304 with a 0.25 mm brass wire diameter. The optimal machining conditions for the wire cutting machining parameters had been predicted to be voltage, pulse-on, pulse-off, and wire feed. The optimal machining performance for the MRR was obtained at a voltage of 50 V (level 1), 6 µs pulse-on time (level 3), 8 µs pulse-off time (level 3), and 7 m/min wire feed rate (level 3) settings. The study demonstrated that voltage is a significant parameter for MRR. In addition to the pulse-on time, ion turned out to be the significant parameter for kerf, and the wire feed rate was the significant parameter for surface roughness. 

Looking at the aforementioned review, a number of experimental studies have attempted to examine the effect of a number of process parameters on the performance of the machining responses of WEDM. Nevertheless, most of the conducted trials either limited the number of process parameters or restricted the range of the values examined. Thus, one can argue that there has been a margin for improvement in obtaining data by conducting an extensive and thorough examination of the effect of a wide range of governing factors of the WEDM process and their levels on the generated surface quality and productivity when processing AISI 304 alloy steel. The study is enriched with a detailed statistical analysis of the obtainable effects of the process parameters and their interactions on the considered responses. In this context, the current study aims to carry out a systematic study to investigate the effect of varying process parameters, such as voltage, feed, pulse-on time, pulse-off time, and current intensity, on the performance of WEDM-processed AISI 304 in terms of material removal rate and surface roughness. The experimental results will be statistically analyzed, allowing us to quantify the individual and interaction effects of the process parameters on each response as a pre-step to optimizing the process afterwards.

The paper is organized as follows. Following this introduction, the experimental work is provided, which entails an explanation of the materials and experimental setup, experimental procedure and process parameters, and characterization techniques utilized to assess the quality marks. Next, the statistical analysis conducted is reported, followed by a discussion of the experimental and statistical results. Finally, conclusions are drawn based on the main findings of the conducted trials.

## 2. Materials and Methods

### 2.1. Materials and Experimental Setup

The experiments were carried out on stainless steel 304 rectangular specimens with the dimensions of 120 mm × 30 mm and a 3.1 mm thickness. The chemical composition [29] and physical properties [30] of stainless steel 304 are listed in Table 1 and Table 2, respectively.

**Figure 3:**
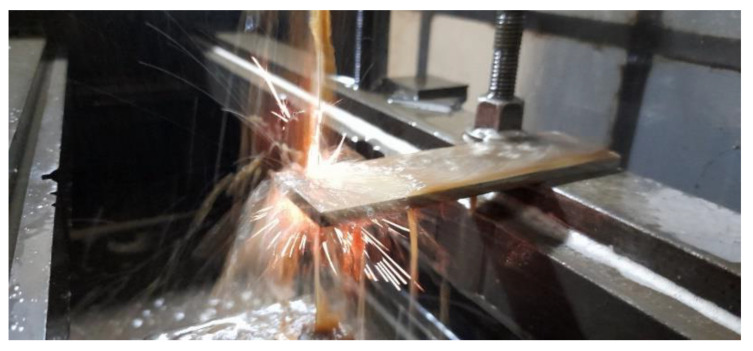
Experimental setup for wire EDM of stainless steel 304.

The machine used was an ONA NX3 EDM with a 1 µm positional resolution. The experiments included molybdenum wires with a diameter of 0.18 mm and a wire tension of 8 (index). As dielectrics, water and gel were used. Prior to the WEDM machining tests, the surface to be machined was ground using a silicon carbide abrasive wheel to ensure that the surfaces of the workpiece were fine and flat and that both were parallel in all experiments as shown in Figure 3.

The WEDM tests were conducted at different levels of voltage, traverse feed, pulse-on time, pulse-off time and current intensity (see Table 3). The design of the experiments was full factorial.

The WEDM performance was assessed in terms of the metal removal rate calculated and surface roughness (Ra). 

### 2.2. Experimental Procedure and Characterization

**Figure 4:**
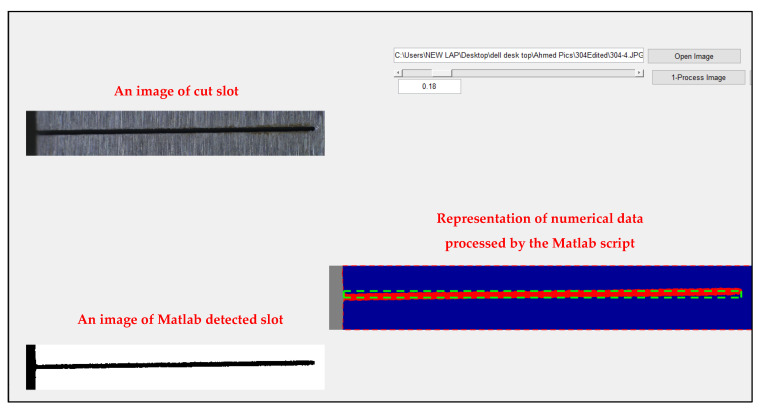
MATLAB processing of WEDM slots.

**Figure 5:**
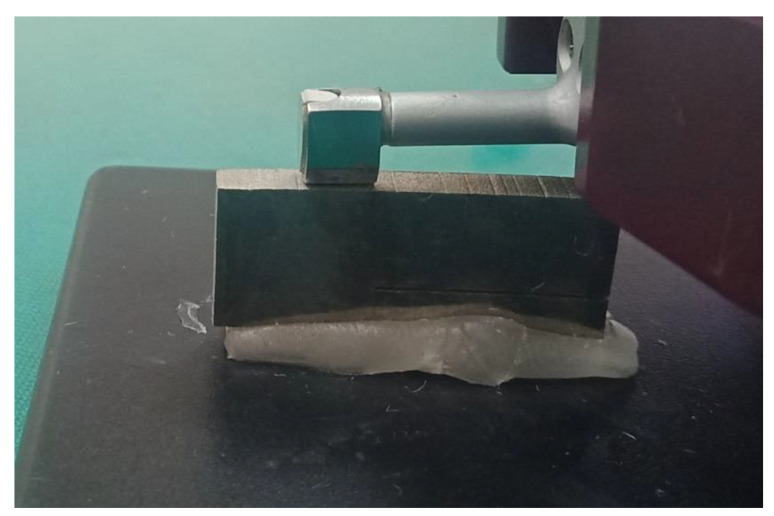
Characterization of surface roughness of cut slots.

Each set of cutting parameters resulted in two cuts; the first with a 15 mm length cut slot and the second with a full-length cut slot of 30 mm. The first one was used to evaluate the slot’s width, while the second cut made it possible to characterize the side walls’ surface roughness. During all tests, different variables were monitored and recorded, such as the machining time and WEDM parameters. Furthermore, the duration of the material removal operation is recorded in each test. The cut slots were captured using a high-resolution camera at the same magnification and focal distance. Following the tests, the collected data and images were analyzed using the MATLAB 2015B package to detect the width of cut slots, as shown in Figure 4, allowing the total material removed and thus the material removal rate to be calculated. To measure the surface roughness of the machined surfaces under the different variables of the WEDM, the surface roughness (Ra) of the walls of the cut slots was characterized using a Mitutoyo SJ-210 surface profilometer from Mitutoyo Corporation (Kawasaki, Japan), as shown in Figure 5, and an average of seven measurements, taken across the entire cut length, were calculated.

### 2.3. Regression Modelling

The correlation between the experimentally observed responses and the input process parameter was defined using statistical regression following a quadratic expression (Equation (1)):

y = b_0_ + ∑b_i_x_i_ + ∑b_ii_x^2^_ii_ + ∑b_ij_x_i_x_j_ (1)

where ‘y’ is the presented response (e.g., MRR in mm^3^/min or surface roughness, Ra in μm), ‘b_0_’,’b_i_’, ‘b_ii_’, and ‘b_ij_’ are the regression coefficients, and ‘x_i_’ and ‘x_j_’ are the ith and jth values of the input parameter, applied voltage, traverse feed, pulse-on time, pulse-off time, and current intensity, in this study. 

Equations (2) and (3), show, respectively, the predicted values of MRR (in mm^3^/min) and surface roughness (in μm) for WEDM of the stainless steel 304 specimens. In the equations, V, f, P_on,_ P_off_, and C are the normalized values of applied voltage, traverse feed, pulse-on time, pulse-off time, and current intensity. The measured values of the process parameters were normalized to [−1,1]. In Equation (2), 

‘MRR’ is the predicted material removal rate;

MRR = 4.9752 + 0.19094 V_n_ + 0.9801 f_n_ + 0.16364 (P_on_)_n_ + 0.29526 C_n_ + 0.085168 V_n_ f_n_
− 0.12767 V_n_ C_n_ − 0.0766 f_n_ (P_off_)_n_ + 0.15747 f_n_ (P_on_)_n_ + 0.11649 f_n_ C_n_ (2)

‘Ra’ is the predicted surface roughness for WEDM;

Ra = 5.0213 + 0.1695 (P_off_)_n_ + 0.41324 (P_on_)_n_ + 0.52206 C_n_ − 0.1832 V_n_ (P_on_)_n_ (3)

These regression models (Equations (2) and (3)) were then used to analyze the effects of the different input parameters, singly and in combination, on the measured surface roughness and calculated material removal rate. These are described below. In addition, an analysis of variance (ANOVA) was used to determine the input parameters with the greatest impact on the responses.

## 3. Results and Discussion 

This section reports the results obtained for the material removal rate and surface roughness followed by a statistical analysis of the experimental results to discuss the interaction effects of the examined parameters on both responses. 

### 3.1. Material Removal Rate

**Figure 6:**
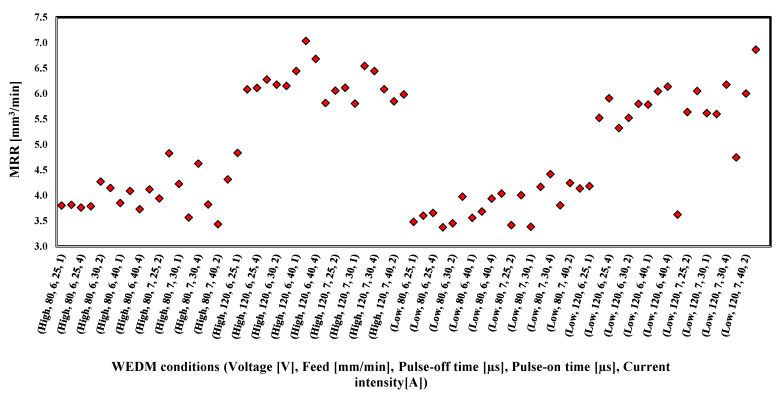
Results of MRR under the whole range of applied process parameters.

Figure 6 illustrates the calculated MRR for the entire range of the applied process parameters. MRR was calculated using the area of the cut slot detected using the MATLAB script multiplied by the thickness of the workpiece. Looking at the MRR results presented, the combination of high feed rate, high current, and high voltage was found to give the highest MRR, while the pulse-off and pulse-on times did not have an effect as significant as the first three parameters.

**Figure 7:**
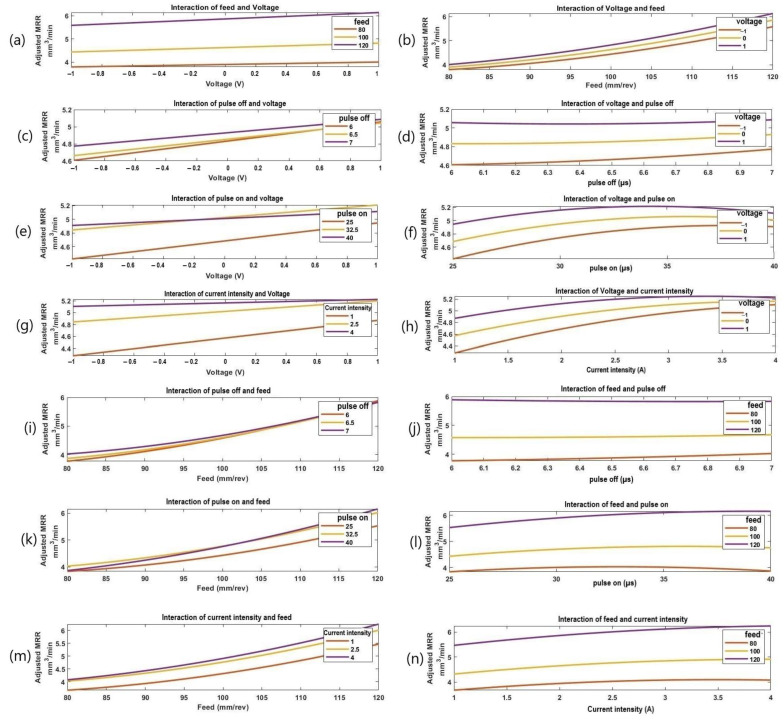

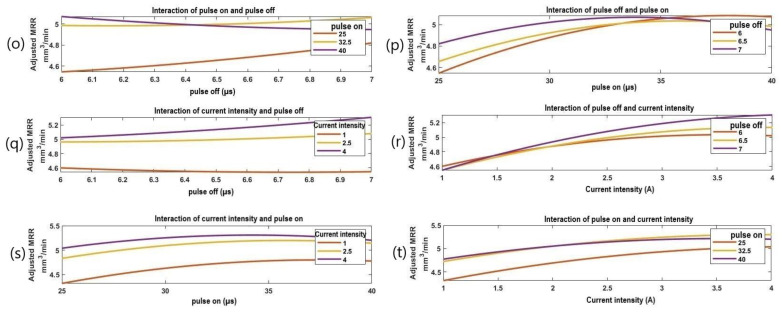
The interactive effects of the process parameters on MRR; (**a**) the effect of voltage at different levels of feed, (**b**) the effect of feed under different levels of voltage, (**c**) the effect of voltage at different levels of pulse-off time, (**d**) the effect of pulse-off time under different levels of voltage, (**e**) the effect of voltage at different levels of pulse-on time, (**f**) the effect of pulse-on time under different voltage, (**g**) the effect of voltage at different levels of current intensity, (**h**) the effect of current intensity under different levels of voltage, (**i**) the effect of feed at different levels of pulse-off time, (**j**) the effect of pulse-off time under different levels of feed, (**k**) the effect of feed at different levels of pulse-on time, (**l**) the effect of pulse-on time under different feed, (**m**) the effect of feed at different levels of current intensity, (**n**) the effect of current intensity under different feed, (**o**) the effect of pulse-off time at different levels of pulse-on time, (**p**) the effect of pulse-on time under different pulse-off time, (**q**) the effect of pulse-off time at different levels of current intensity, (**r**) the effect of current intensity under different pulse-off time, (**s**) the effect of pulse-on time at different levels of current intensity, and (**t**) the effect of current intensity under different pulse-on time.

Figure 7 illustrates the interaction effect plots of the process parameters (voltage, feed, pulse-on time, pulse-off time, and current intensity) on the calculated MRR. These graphs were plotted using the regression models presented in Equation (2). Looking at the results, it is obvious to observe the significant proportional effect of the feed on the MRR, while the applied voltage has shown more significant effect at high feed. Figure 7 shows the impact of machining variables on MRR. In particular, as shown in Figure 7a,b, MRR increases with the increasing voltage at different values of feed. At high feeds, the voltage has a greater impact on MRR than at low feeds. The increased discharge gap between the workpiece and wire electrode causes the rate of material removal to rise when the voltage is raised. The spark’s strength and intensity decrease as the gap increases. The intensity of the spark generated during the transformation from the wire to the workpiece is lost in the medium due to the high dielectric fluid strength. From Figure 7c,d, the MRR gradually increases with the increasing voltage and pulse-off time. At low pulse-off times, the voltage had a greater influence on MRR than at high pulse-off times. The reason why MRR increases as the pulse-off time increases is because very low Toff values might lead to wire breakage and unstable discharge conditions. When sparking becomes unstable, it is preferable to raise Toff, as this enables a lower pulse duty factor, which lowers the average gap current.

The effect of pulse-on time and voltage on MRR is shown in Figure 7e,f. It can be seen that the MRR gradually rises as the voltage rises; see Figure 7e. In addition, for all voltage values, there was a relatively rapid increase in MRR with the increase in pulse-on time. The reason for this is that throughout this time, a voltage has been applied across the wire electrode. The cutting rate increases as Ton increases because a single pulse’s discharge energy rises. According to Figure 7g,h, for various current levels, the material removal rate increases with the increasing voltage. Voltage had a greater impact on MRR at low current intensity than at a high current intensity. This is because the energy of the pulse discharge increases as the peak current increases. The gap state may become unstable when the Ton, Toff, voltage, and feed parameters are not appropriately mixed at greater amounts of peak current. Additionally, by reducing the peak current value, a steady discharge condition can be achieved. The effect of feed and pulse-off time on MRR is shown in Figure 7i,j. The MRR is in proportion to the feed at the different values of the pulse-off time. At high feeds, the pulse-off time had a lower impact on MRR than at low feeds, which agree with [24]. From Figure 7k,l the MRR gradually increased with the increasing feed and pulse-on time. Pulse-on time had a higher influence on MRR at higher feeds than lower feeds. Figure 7m,n, shows how current and feed affect MRR. Figure 7m illustrates how the MRR increases progressively as the feed increases. Additionally, in line with what was reported in [27,31], with the increase in current, the MRR for all feed levels saw a slight increase. Figure 7o,p show that the material removal rate increases with the increasing pulse-off time for different pulse-on time values. MRR was significantly influenced by the pulse-off time at a low pulse-on time than at a high pulse-on time. These results are in quite agreement with [32]. Figure 7q,r shows how the value of the pulse-off time and current enhances the rate of the material removal. At greater pulse-off time levels than at lower levels, the current had a noticeable impact. Figure 7s,t shows the impact of the pulse rate on time and current on MRR. Figure 7s shows how the MRR gradually increases as the pulse-on time increases, which is in good agreement with those reported in [24,27]. Additionally, for all current levels, the MRR increased quite quickly as the pulse-on time increased. At a low pulse-on time, the current intensity had a greater effect on MRR than at a high pulse-on time.

**Figure 8:**
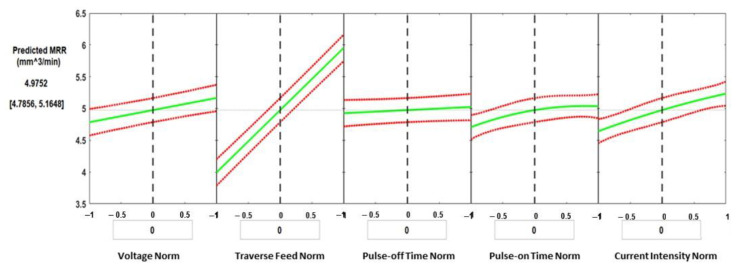
Prediction plots created using the developed process regression model for the MRR.

An ANOVA analysis of WEDM showed that the most significant parameter affecting the material removal rate was the feed (*p*-value = 9.72 × 10^−29^), followed by the current intensity (*p*-value = 6.02 × 10^−7^), and the voltage (*p*-value = 3.77 × 10^−5^). The MATLAB regression model was developed in order to generate prediction plots as presented in Figure 8. Predicted plots were used to show the main effects of each individual process parameter when all other process parameters were kept constant. The slope of the effects of the five parameters shows that the feed is the most significant parameter, which agrees with the ANOVA results with a *p*-value of 9.72 × 10^−29^. Applied current intensity and voltage came in as the second and third significant parameters, with *p*-values of 6.02 × 10^−7^ and 3.77 × 10^−5^, respectively. The response prediction for a change in the normalized value of the process parameters is shown by the green line in each plot. The 95% confidence bounds for the expected response value are shown by the dashed red curves. The vertical dashed line can be moved along the trends to the corresponding parameter values to obtain the predicted response. This can be used to find the process parameters that give the lowest and highest values of material removal rates. For the given process parameters, the predicted material removal rate is 4.9752 mm^3^/min, and the corresponding normalized values of the process parameters are: voltage = 0 V, feed = 100 mm/min, pulse-off time = 6.5 µs, pulse-on time = 30 µs, and current intensity = 2 A.

### 3.2. Surface Roughness

**Figure 9:**
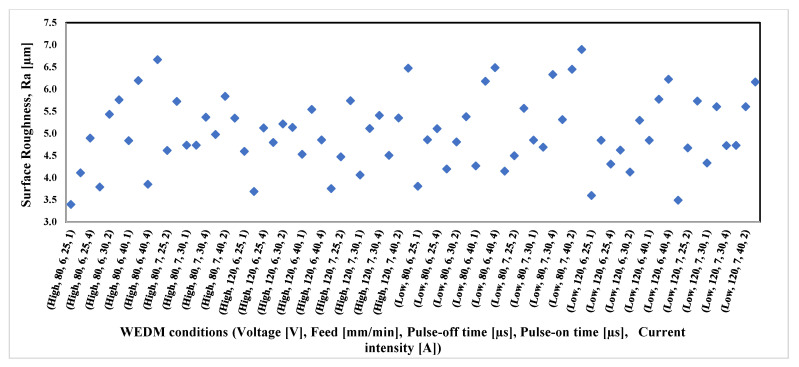
Results of surface roughness at the whole range of applied process parameters.

Figure 9 shows the surface roughness measured at the entire range of process parameters. No clear trends were observed for the effect of the process parameters on the generated surface roughness. 

**Figure 10:**
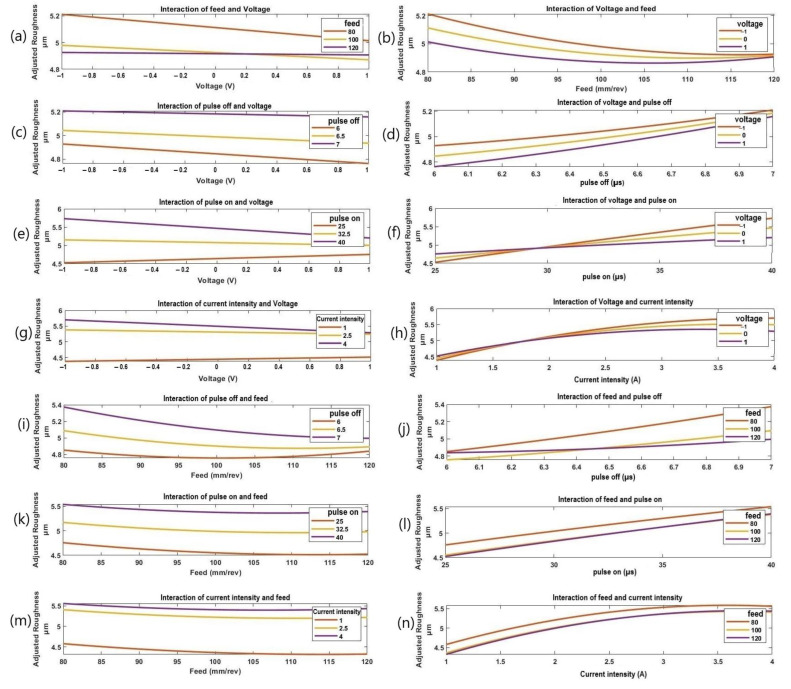

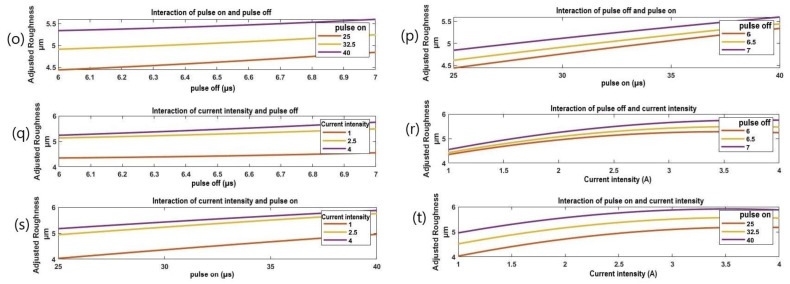
The interactive effects of process parameters on Ra; (**a**) the effect of voltage at different levels of feed, (**b**) the effect of feed under different levels of voltage, (**c**) the effect of voltage at different levels of pulse-off time, (**d**) the effect of pulse-off time under different levels of voltage, (**e**) the effect of voltage at different levels of pulse-on time, (**f**) the effect of pulse-on time under different voltage, (**g**) the effect of voltage at different levels of current intensity, (**h**) the effect of current intensity under different levels of voltage, (**i**) the effect of feed at different levels of pulse-off time, (**j**) the effect of pulse-off time under different levels of feed, (**k**) the effect of feed at different levels of pulse-on time, (**l**) the effect of pulse-on time under different feed, (**m**) the effect of feed at different levels of current intensity, (**n**) the effect of current intensity under different feed, (**o**) the effect of pulse-off time at different levels of pulse-on time, (**p**) the effect of pulse-on time under different pulse-off time, (**q**) the effect of pulse-off time at different levels of current intensity, (**r**) the effect of current intensity under different pulse-off time, (**s**) the effect of pulse-on time at different levels of current intensity, and (**t**) the effect of current intensity under different pulse-on time.

Figure 10 shows the impact of the machining variables on the surface roughness (Ra). In particular, as shown in Figure 10a,b, surface roughness decreased with the increasing voltage at different feed rates. At low feeds, the voltage had a greater impact on the Ra than at high feeds. When the gap voltage is low, the feed speed is too high, which can easily allow a short circuit to occur and prevents the discharge of the products of electric erosion, leading to unstable discharge processing and low cutting speed. This causes the surface roughness to decrease as the voltage is increased. Given that a lot of discharge energy is focused on the cutting surface and that sticky material still adheres to the cutting surface, surface roughness (Ra) decreases with increasing voltage and pulse-off time, as shown in Figure 10c,d. These results agree with those reported in [28]. Voltage had a higher influence on Ra at the low pulse-off time than at the high pulse-off time. With a large value of T_off_, there were fewer discharges in a given time, resulting in the formation of shallow crates on the workpiece surface, which might be the explanation for the reduction of the Ra with an increasing pulse-off time. Therefore, the SR decreases, which is in a good agreement with the results in [32]. Wire breakage occurred while using extremely low T_off_ values. The effect of the pulse-on time and voltage on the Ra is shown in Figure 10e,f. It can be seen that the Ra decreases as the voltage rises (see Figure 10e). In addition (see Figure 10f), it was noticed that with increasing voltage values, a slight increase in surface roughness was observed with the increasing pulse-on time from 25 µs to 29 µs followed by a decrease in the surface roughness when the pulse-on time rose from 29 µs to 40 µs. This is explained by the fact that a higher T_ON_ makes the cutting zone’s available discharge energy larger, which causes deep craters to occur on the workpiece’s surface and raises the SR. According to Figure 10g,h, for various current intensities, the surface roughness decreases with an increasing voltage. The voltage had a greater effect on the Ra at a high current intensity than at a low current intensity. In addition, it was noticed that with the increasing voltage values, a slight increase in the surface roughness was observed with an increasing current intensity from 1 A to 1.75 A, followed by a decrease in the surface roughness when current intensity rose from 1.75 A to 4 A. The explanation is that when peak current increases, it also increases the electric energy of a single pulse discharge. When the peak current rises, the cutting speed increases, and the surface roughness somewhat worsens. This process is steady, and there are no obstructions to the electric erosion products’ discharge. Cutting speed increased quickly as a result of an increase in the average discharge energy per unit time, and as the diameter of the discharge pit on the cutting surface grew, increased the roughness of the surface. The effect of feed and pulse-off time on surface roughness is shown in Figure 10i,j. The Ra decreases with the increasing feed at different values of pulse-off time. At low feeds, the pulse-off time had a higher impact on the Ra than at high feeds.

According to Figure 10k, the Ra gradually decreases with the increasing feed while increasing with an increasing pulse-on time. Pulse-on time had nearly the same influence on the Ra at low and high feeds. These results agree with [27]. Figure 10m,n shows how the current intensity and feed affect the Ra. Figure 10m illustrates how the Ra decreases progressively as the feed increases. Additionally, with the increase in current intensity, Ra for all feed levels saw a slight increase. At a low current intensity, the feed had a greater impact on Ra than at a high current intensity, which agrees with [27]. Figure 10o,p show that the surface roughness increases with an increasing pulse-off time for different pulse-on time values. Ra was significantly influenced by the pulse-off time at a low pulse-on time than at a high pulse-on time. Figure 10q,r, shows how the value of the pulse-off time and current intensity enhances the surface roughness. At greater current intensity levels than at lower levels, the pulse-off time has a noticeable impact. Figure 10s,t depicts the effect of the pulse-on time and the current intensity on the Ra. Figure 10s shows how the Ra gradually increases as the pulse-on time increases for all current intensity levels. The pulse-on time had a greater effect on Ra at low current intensity compared to high current intensity (see Figure 10t).

**Figure 11:**
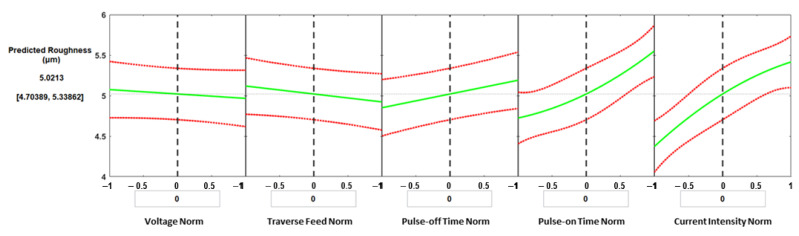
Prediction plots for the surface roughness.

ANOVA analysis for WEDM showed that the most significant parameter affecting the surface roughness was the current intensity (*p*-value = 1.89 × 10^−7^), followed by pulse-on time (1.602 × 10^−5^), and pulse-off time (0.0204). Again, the prediction plots were developed by the MATLAB regression model, as presented in Figure 11. The slope of the effect of the five parameters shows that the current intensity is the most significant parameter, which agrees with the ANOVA results with a *p*-value of 1.89 × 10^−7^. Applied pulse-on time and pulse-off time were the second and third significant parameters, with *p*-values of 1.602 × 10^−5^ and 0.0204, respectively. For the given process parameters, the predicted surface roughness is 5.0213 µm, and the corresponding normalized values of the process parameters were: voltage = 0 V, feed = 100 mm/min, pulse-off time = 6.5 µs, pulse-on time = 30 µs, and current intensity = 2 A. 

## 4. Conclusions

The findings of the experimental investigation into how process parameters affect the material removal rate and surface roughness of stainless steel 304 when cut with a wire EDM are presented in this paper. On the basis of experimental and statistical research, the WEDM’s machining performance has been examined. Molybdenum wires were used in these experiments, and the following process variables were used: the applied voltage, the traverse feed, the pulse-on time, the pulse-off time, and the current intensity. As machining outputs, surface roughness and MRR were evaluated. The cut slots’ shape was characterized using a MATLAB image processing toolbox. The main conclusions are as follows:

- For the WEDM of stainless steel 304, an increase in MRR was observed with increasing applied voltage, feed, pulse-on time, and current intensity.
- The results show that the feed is the most significant parameter on MRR, which agrees with the ANOVA results with a *p*-value of 9.72 × 10^−29^. Applied current intensity and voltage came second and third as the most significant parameters, with *p*-values of 6.02 × 10^−7^ and 3.77 × 10^−5^, respectively.
- Pulse-on time was found to have a higher impact on MRR at higher feed levels than lower feeds, whereas pulse-off time has less of an impact at high feeds than low feeds.
- For all feed levels, MRR increases slightly with an increase in current intensity; MRR also increases with the increase in the pulse-off time for various pulse-on time levels.
- The results of the effect of the five parameters on obtainable surface roughness show that the current intensity is the most significant parameter, which agrees with the ANOVA results with *p*-value of 1.89 × 10^−7^. Applied pulse-on time and pulse-off time came second and third significant parameters with *p*-values of 1.602 × 10^−5^ and 0.0204, respectively.
- Ra slightly increases as current intensity and pulse-on time increase from 25 to 29 µs and from 1 to 1.75 A, respectively, followed by a decrease in the surface roughness with the increase in current intensity and pulse-on time from 29 to 40 µs and 1.75 to 4 A, respectively.
- There is trade-off between the effect of the process parameters on both responses, material removal rate, and surface roughness, which indicates the challenge to optimize both responses simultaneously. This clearly demonstrates the need for a reliable multi-objective optimization technique to obtain sets of working conditions for both high MRR and low surface roughness together.

## Figures and Tables

**Table 1:** Material composition of SS 304 [29].

Grad	Mn	C	S	P	Si	Ni	N	Cr	Fe
Stainless Steel 304	2.00	0.08	0.03	0.045	0.75	8	0.10	18–20	Balance

**Table 2:** Physical properties for 304 stainless steel alloys sheet [30].

Property	Value
Density	8.00 g/cm^3^
Melting Point	1450 °C
Modulus of Elasticity	193 GPa
Electrical Resistivity	0.072 ×10^−6^ Ω.m
Thermal Conductivity	16.2 W/m.K at 100 °C
Thermal Expansion	17.2 × 10^−6^/K at 100 °C

**Table 3:** WEDM conditions used for experiments.

WEDM Condition (Unit)	Levels
Voltage, V (V)	High, Low
Traverse feed, f (mm/min)	80, 120
Pulse-on time, P_on_ (µs)	25, 30, 40
Pulse-off time, P_off_ (µs)	6, 7
Current intensity, C (A)	1, 2, 4

## Data Availability

Not applicable.
